# Debridement, Antibiotics, and Implant Retention for Acute Periprosthetic Joint Infection

**DOI:** 10.1111/os.12641

**Published:** 2020-03-11

**Authors:** Chao‐fan Zhang, Long He, Xin‐yu Fang, Zi‐da Huang, Guo‐chang Bai, Wen‐bo Li, Wen‐ming Zhang

**Affiliations:** ^1^ Department of Orthopaedic Surgery The First Affiliated Hospital of Fujian Medical University Fuzhou China; ^2^ Department of Hand Surgery The Second Hospital of Fuzhou Affiliated to Xiamen University Fuzhou China

**Keywords:** Acute periprosthetic joint infection, Debridement, Implant retention, Total hip replacement, Total knee replacement

## Abstract

**Objective:**

To report on our clinical outcomes and on the experience of managing acute periprosthetic joint infection (PJI) with debridement, antibiotics, and implant retention (DAIR).

**Methods:**

We performed a retrospective review of all patients who were diagnosed with acute PJI after primary hip or knee replacement surgeries and who were managed with DAIR in our prospective joint replacement registry from 2008 to 2019. The diagnosis of PJI was made according to the 2011 Musculoskeletal Infection Society (MSIS) criteria. The symptom onset duration, inflammatory marker levels (i.e. C‐reactive protein [CRP], erythrocyte sedimentation rate [ESR], white cell count [WBC], and synovial WBC count), functional scores including the Knee Society Score (KSS), the KSS functional score and the Harris Hip Score (HHS), bacteriology, and surgical outcomes of the patients were tracked and recorded. A paired sample of joint fluid and tissues was also sent for a metagenomic next‐generation sequencing (mNGS) test. A paired‐samples *t*‐test was used to compare the differences in the inflammatory markers and functional scores before and after surgery.

**Results:**

A total of 24 patients with 7 infections after hip replacements and 17 infections after knee replacements were included. A total of 21 patients exhibited early postoperative infections, and 3 exhibited late acute hematogenous infections. During a mean follow‐up time of 29.2 ± 15.1 months, 22 patients were successfully treated, whereas 2 patients were unsuccessfully treated and required repeated DAIR. The overall success rate of DAIR was 91.7%. For staphylococcal infections, DAIR had a 100% success rate. Five patients who presented with symptoms between 4 and 8 weeks also achieved a 100% success rate. At the last follow‐up, the mean CRP level decreased from 52.6 ± 34.0 to 5.4 ± 3.5 (*P* < 0.001), and the mean ESR level decreased from 72.3 ± 34.3 to 20.2 ± 12.1 (*P* < 0.001). The mean KSS score increased from 44.8 ± 12.2 to 81.4 ± 9.2 (*P* < 0.001), and the mean KSS functional score increased from 38.1 ± 3.5 to 73.9 ± 23.0 (*P* < 0.001), and the mean HHS score increased from 34.4 ± 6.9 to 84.1 ± 15.1 (*P* < 0.001). The overall pathogen identification rate was 91.7% (22/24 cases). The success rates for *Staphylococcus*, *Streptococcus*, and the other pathogens were 100% (9/9 cases), 71.4% (5/7 cases), and 100% (6/6 cases), respectively.

**Conclusion:**

Debridement, antibiotics, and implant retention has a high success rate for the treatment of acute PJI and may be performed in selected patients whose symptoms have been sustained for over 4 weeks. A high rate of success for staphylococcal infections was reported with the use of DAIR.

## Introduction

Periprosthetic joint infection (PJI) after primary total hip and total knee arthroplasties is a rare, but catastrophic complication that can cause significant morbidity to the patient and cost to the healthcare system^1^. It was found from the Medicare inpatient dataset that the 1‐year and 5‐year risk of PJI was 0.69% and 1.09% for total hip arthroplasty and 0.74% and 1.38% for TKA, respectively^2^. Although not commonly seen, treating PJI has already been estimated to cost the US$566m in 2009, and the figure is projected to reach $1.62bn in 2020^1^. It has to be noted that these numbers seem to be underestimated because many asymptomatic infections do not come to the attention of the surgeon.

Acute PJI, as proposed by Tsukayama^3^, are defined as infections that occur within 4 weeks from the primary arthroplasty or as an acute onset of symptoms within 4 weeks in a previously well‐functioning joint (a hematogenous infection). Management options for acute PJI basically include antibiotic suppression and surgical management, which includes open debridement, antibiotics, and implant retention (DAIR), arthroscopic debridement, one‐stage revision, and two‐stage revision. In the past few decades, DAIR, which primarily includes a thorough debridement and exchange of the polyethylene inserts or femoral head, has been widely accepted as the first‐line treatment for acute PJI. Surgical debridement is a less invasive and less expensive procedure compared to one‐stage or two‐stage revision, and polyethylene insert exchange theoretically allows the surgeon to gain better access to the posterior joint capsule and bilateral gutters for radical debridement.

However, the efficacy of DAIR remains unclear, as the literature has demonstrated variable success rates, ranging from 26% to 92%^2^, ^3^, ^4^, ^5^, ^6^, ^7^, ^8^, ^9^, ^10^, ^11^, ^12^. Several risk factors have been proposed to affect the outcomes of DAIR, including patient age, the American Society of Anesthesiologists (ASA) score, diabetes mellitus (DM), rheumatoid arthritis, and steroid therapy^4^. In addition, the roles of the optimal timing of DAIR treatment, antibiotic regimens, the involved microorganism, and the decision of whether to exchange the polyliner are still unclear. Several authors have also questioned whether DAIR can be performed in patients whose symptoms have been present for over 4 weeks^5^.

From our clinical experience, we believe that DAIR, if performed correctly, can have high success rates for patients with acute PJI. Most patients who have been admitted to our team of physicians had high infection eradication rates and satisfactory functional outcomes. Therefore, the purpose of this study was: (i) to retrospectively review the patients diagnosed with acute PJI and managed with DAIR, and report on our treatment success rate and analyze the key factors that determine the success rate; (ii) to explore if DAIR can be performed in patients whose symptoms have been sustained for over 4 weeks; and (iii) to investigate if DAIR can be performed in patients with staphylococcal infections. We had three hypotheses: (i) DAIR has a high success rate for the treatment of acute PJI; (ii) DAIR can be performed in patients with delayed PJI (i.e. patients with symptoms that have persisted for over 4 weeks); and (iii) DAIR has a high success rate for acute staphylococcal infections.

## Methods

### *Study Characteristics*

This study was approved by the Ethics Committee and by the Institutional Review Board of our institution (Approval No: [2019] 202). Informed consent was obtained from each patient before the data were collected. A retrospective review of our prospective joint replacement registry from 2008 to 2019 was performed.

### *Inclusion and Exclusion Criteria*

The inclusion criteria included:

(i) Patients who were diagnosed with acute PJI after hip or knee replacement surgeries. The diagnosis of PJI was based on the 2011 Musculoskeletal Infection Society (MSIS) PJI diagnostic criteria^6^. Both early postoperative infections (Tsukayama type 2) and late hematogenous infections (Tsukayama type 3) were included^3^. “Acute” infections were defined as infections that occurred within 3 months after the primary surgery, or in which the onset of symptoms occurred within 3 months in a well‐functioning joint, as was proposed by Zimmerli^7^.

(ii) Patients who were managed with DAIR.

(iii) The major evaluation indicators included age, preoperative and postoperative C‐reactive protein (CRP) levels, erythrocyte sedimentation rate (ESR) levels, white blood cell (WBC) counts, lymphocyte counts, bacteriology and synovial total cell counts.

The exclusion criteria included:

(i) Infections that occurred beyond 3 months after the primary surgery, or in which the onset of symptoms occurred beyond 3 months in a well‐functioning joint.

(ii) Infections managed with one‐stage or two‐stage revision surgery.

(iii) Patients with incomplete follow‐up records.

### *Surgical Technique*

Our center is a tertiary referral center in Fujian Province, which has a population of 38.6 mn individuals. Most of the patients had received joint replacement surgeries in other hospitals but were transferred to our center for further management. Once a patient was diagnosed with acute PJI, empirical antibiotics were used, and DAIR was performed as soon as possible, regardless of the microorganism culture results. Surgery was performed by a single senior surgeon (W. Zhang) using a standard operating setup.

In general, spinal anesthesia was performed. The surgical site was disinfected three times with a tincture of iodine, followed by 70% alcohol. A previous incision was used, and the superficial wound was debrided.

Before opening up the synovial cavity, aspiration was again performed to collect synovial fluid, and the fluid was sent for either culturing or for a metagenomic next‐generation sequencing (mNGS) test. Once the cavity was cut open, at least five separated periprosthetic tissue samples were collected with the use of a surgical knife cut, and the samples were sent for microbiological culture and mNGS.

The polyethylene insert or femoral head was then removed, and systematic debridement was performed to remove all of the infected or necrotic tissues. The wound was soaked in iodine solution for 30 min and irrigated with 3 L of saline using a pulsed lavage gun. The wound was temporally closed and re‐draped, and a new set of surgical tools was used.

After re‐disinfecting the surgical site, the new insert/femoral head was inserted, and the wound was closed with a three‐layer closure. The drain was typically kept in place for 2 days. A tourniquet was used if too much bleeding was encountered during the knee debridement.

### *Microbial Culture*

Culturing has also been used as a standardized procedure in our center. Joint fluid, once collected, was injected into aerobic and anaerobic bottles, after which it was inoculated onto fungal plates. A small amount of fluid was also injected into a pediatric blood culture bottle. Tissues were collected using a blade cut (instead of using an electrode) and further cut into pieces, before being stored in sterile containers and immediately sent for culturing (from the operation theater to the Department of Microbiology) by designated personnel. Culturing was routinely performed for 7 days; however, in cases of negative results or cases of suspected low‐virulence pathogens, culturing was prolonged to 14 days. A paired sample of joint fluid and tissues was also sent for an mNGS exam, which was performed by the BGI Group (Shenzhen, China). The results were normally available in 48 hours.

### *Antibiotic Regimen*

An empirical antibiotic regimen of vancomycin (1.0 g ivgtt q12 h) combined with ceftazidime (2.0 g ivgtt q8 h) was prescribed until the culture/mNGS results were available. This regimen was then changed to pathogen‐specific antibiotics, according to the drug susceptibility results. In certain circumstances, consultation from the specialists from the Department of Infectious Diseases was ordered. The liver and renal function were routinely and strictly monitored. In general circumstances, intravenous (IV) antibiotics were used for 2 weeks, followed by the use of oral antibiotics for an additional 4 weeks.

### *Outcome Measurement*

The surgical outcomes were defined as being successful if the patients' clinical symptoms had been relieved, if the inflammatory marker levels (including CRP, ESR, and WBC counts) had returned to normal, if the X‐rays showed no prosthetic loosening, and if no lifelong suppression of antibiotics was required, at a minimum of 1‐year follow‐up. The outcomes were defined as failed if the patients required any further surgeries (e.g. additional DAIR or any forms of debridement, or one‐stage or two‐stage revisions) or if they required lifelong suppression of antibiotics.

#### *Inflammatory Makers*

Once an infection was suspected, blood tests of inflammatory markers (including CRP, ESR, and WBC tests) were ordered. A joint aspiration was performed to collect the synovial fluid, and the fluid was sent for WBC and differential count tests, as well as for a microbiological culture. For hip infections, aspiration was performed under ultrasound guidance. After discharge, patients were followed routinely with 3‐month intervals, and CRP, ESR, and WBC tests were ordered at each visit. All these tests were performed by our colleagues from the Department of Laboratory Medicine.

#### *Knee Society Scores*

The Knee Society Score (KSS) is designed to provide a simple and objective scoring system to rate the knee and the patient's functional abilities before and after TKA. It has a “Knee Score” section, which includes pain (6 items, 0–50 points), flexion contracture (5 items, 0–15 points), extension lag (4 items, 0–15 points), total range of flexion (25 items, 0–24 points), alignment (12 items, 0–20 points), antero–posterior stability (3 items, 0–10 points), and mediolateral stability (4 items, 0–15 points). The “Functional Score” includes three sections: walking (6 items, 0–50 points), stairs (5 items, 0–50 points), and walking aids used (4 items, 0–20 points). Both sections are scored from 0 to 100, with lower scores being indicative of worse knee conditions and higher scores being indicative of better knee conditions^8^. The KSS score and the KSS functional score, were evaluated by the designated residents before the DAIR surgery and at each postoperative follow‐up.

#### *Harris Hip Score*

The Harris Hip Score (HHS) was designed to be a standardized assessment of patients following THA. It is a physician‐completed instrument that consists of subscales for pain severity (1 item, 0–44 points), function (7 items, 0–47 points), absence of deformity (1 item, 0–4 points), and range of motion (2 items, 0–5 points). Scores range from 0 (worse disability) to 100 (less disability)^9^. The HHS score for patients with hip infections was evaluated by the designated residents before the DAIR surgery and at each postoperative follow up.

### *Statistical Analysis*

A paired‐samples *t*‐test (for the parametric data) was used to compare the differences of the inflammatory markers as well as functional scores before and after surgery by using SPSS software (v22.0, IBM, USA). Statistical significance was demonstrated if the *P*‐value was less than 0.05.

## Results

### *General Results*

A total of 27 patients were tracked from our registry. One patient died from gastric cancer at 18 months after surgery, and 2 patients were lost to follow‐up. These patients were excluded from the study, thus leaving 24 patients who were included in the final analysis. A total of 13 patients were male and 11 patients were female, with a mean age of 63.8 ± 13.3 years and a mean BMI of 25.6 ± 3.5. Seven patients had infections after hip replacements, and 17 patients had infections after knee replacements. The hip replacements (all of which were total hip arthroplasties) were performed for the treatment of various conditions, including 2 femoral neck fractures, 3 cases of osteonecrosis of the femoral head (ONFH), and 1 case of ankylosing spondylitis (AS). For the knee replacement surgeries, 16 cases were TKA and one case was a unicondylar knee replacement (UKA). All of the replacements were performed for knee OA. A total of 21 cases involved early postoperative infections, and three cases involved hematogenous infections. In terms of comorbidities, 18 patients had hypertension, 7 patients had DM, 2 patients had AS, 2 patients had gout, 1 patient had hepatic cirrhosis, and 1 patient had varicose veins.

For the presentations, 2 patients had sinus tracts, and the other patients primarily complained of pain, swelling or fever. The mean symptom onset time was 21.5 days. Five patients presented with symptoms for over 4 weeks but were still managed with the use of DAIR. The mean synovial WBC count was 20,107.5*10^6/L, and the percentage of polymorphonuclear neutrophils (PMN) was 85.3% ± 7.2%. For all the DAIR treatments, the polyethylene insert/femoral head was exchanged during surgery.

### *Success Rate of Debridement, Antibiotics, and Implant Retention*

The mean follow‐up time was 29.2 ± 15.1 months. Twenty‐two cases were successful, and two cases failed. The overall success rate was 91.7%. It was noted that, in the 19 cases in which DAIR was performed within 4 weeks, 17 cases were successful, with a success rate of 89.4% (17/19 cases). In the other 5 cases, the patients had sustained symptoms between 4 and 8 weeks, with an average duration of 43.4 days. These patients still received DAIR and achieved a 100% success rate. For the two failed cases, both patients received repeated DAIR treatments, and no recurrent infections were observed. We observed no complications for the successful cases.

### *Change of Inflammatory Markers*

At the last follow‐up, the levels of inflammatory markers, including CRP and ESR, were significantly decreased, with CRP from 52.6 ± 34.0 to 5.4 ± 3.5 (change percentage [70.0 ± 10.6]%, *P* < 0.001) and ESR from 72.3 ± 34.3 to 20.2 ± 12.1 (change percentage [50.0 ± 14.4]%, *P* < 0.001), respectively (Table 1).

**Table 1:** Changes in inflammatory marker levels and functional scores before DAIR and at the last follow‐up

Parameters	Before DAIR (mg/L)	At the last follow‐up (mm/h)	Percentage change (%)	*P‐*value
CRP	52.6 ± 34.0	5.4 ± 3.5	70.0 ± 10.6	*P* < 0.001
ESR	72.3 ± 34.3	20.2 ± 12.1	50.0 ± 14.4	*P* < 0.001
KSS	44.8 ± 12.2	81.4 ± 9.2	80.7 ± 11.4	*P* < 0.001
KSS functional score	38.1 ± 3.5	73.9 ± 23.0	93.5 ± 18.8	*P* < 0.001
HHS	34.4 ± 6.9	84.1 ± 15.1	140.2 ± 14.6	*P* < 0.001

A paired‐sample *t*‐test was used. Statistical significance was assumed if the *P*‐value was less than 0.05. CRP, C‐reactive protein; DAIR, debridement, antibiotics, and implant retention; ESR, erythrocyte sedimentation rate; HHS: Harris Hip Score; KSS, Knee Society Score.

### *Change of the Knee Society Score and the Knee Society Functional Score*

At the last follow‐up, the functional scores for knee infections, including the KSS and the KSS functional score, were significantly improved, with the KSS from 44.8 ± 12.2 to 81.4 ± 9.2 (change percentage [80.7 ± 11.4]%, *P* < 0.001) and the functional score from 38.1 ± 3.5 to 73.9 ± 23.0 (change percentage [93.5 ± 18.8]%, *P* < 0.001), respectively (Table 1).

### *Change of Harris Hip Score*

At the last follow‐up, the HHS score for hip infections was also significantly improved from 34.4 ± 6.9 to 84.1 ± 15.1 (change percentage [140.2 ± 14.6]%, *P* < 0.001) (Table 1).

### *Bacteriology*

For the bacteriology, cultures were positive in 21 cases (87.5%). There were 9 ethicillin‐resistant *Staphylococcus epidermidis* (MRSE) cases, 3 *Staphylococcus aureus* cases, 3 *Streptococcus agalactiae* cases, 1 *Staphylococcus hemolyticus* case, 1 *Streptococcus agalactiae subsp. equisimilis* case, 1 *Streptococcus gordonii* case, 1 *Bacteroides* case, 1 *Enterococcus faecalis* case, and 1 *Klebsiella pneumonia* case. mNGS tests were performed in 10 cases with all of the positive results. Six cases yielded exactly the same pathogen incidences as were observed in the cultures, but 2 cases yielded completely different incidences. In 1 case, the culture identified Ak *lebsiella pneumoniae* infection, but the mNGS test also identified incidences of *Bacteroides* and *Pseudomonas aeruginosa*. In another case, the culture was negative, but the mNGS test identified a *streptococcus agalactiae* infection (Table 2).

**Table 2:** Pathogen identification results between microbiological culture and mNGS tests

Case number	Culture	mNGS
1	*Staphylococcus aureus*	*Staphylococcus aureus*
2	*Staphylococcus aureus*	Human mycoplasma
3	*Staphylococcus aureus*	*Staphylococcus aureus*
4	*Streptococcus agalactiae*	*Streptococcus agalactiae*
5	*Streptococcus agalactiae*	*Streptococcus agalactiae*
6	*Staphylococcus heemolyticus*	*Staphylococcus haemolyticus*
7	*Bacteroides*	*Bacteroides*
8	*Enterococcus faecalis*	*Enterobacter cloacae*
9	*Klebsiella pneumoniae*	*Klebsiella pneumoniae* + *Bacteroides* + *Pseudomonas aeruginosa*
10	Negative	*Streptococcus agalactiae*

mNGS, metagenomic next‐generation sequencing.

**Figure 1:**
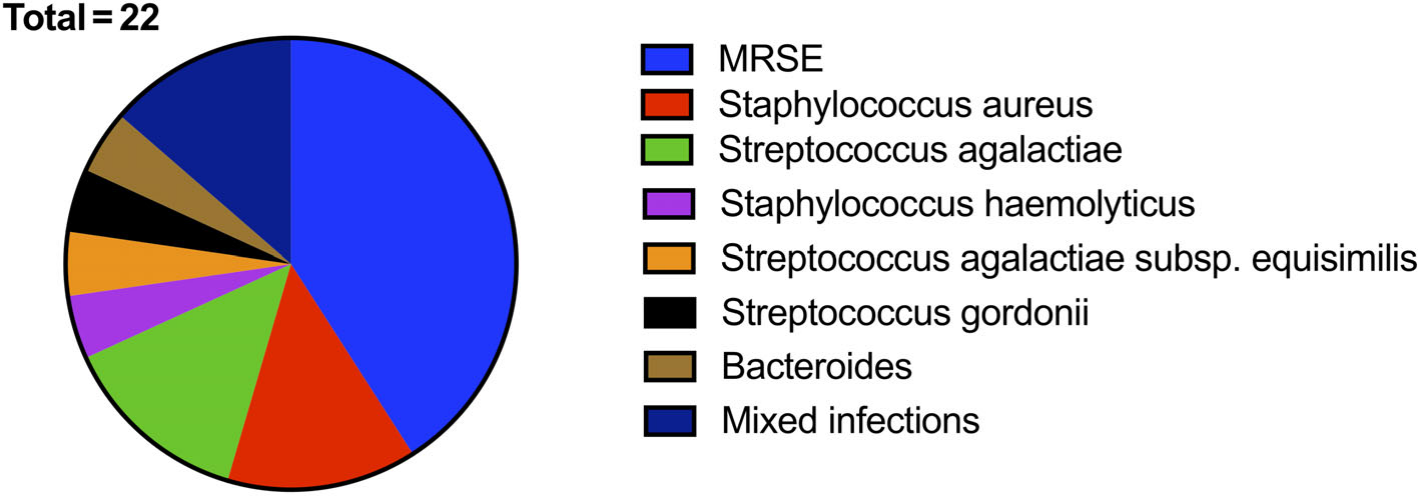
Bacteriology of included cases with positive culture/mNGS results. Cultures were positive in 21 cases (87.5%) and mNGS tests were performed in 10 cases, with all results being positive. In 1 case, the culture was negative, but the mNGS test identified a *Streptococcus agalactiae* infection. The overall pathogen identification rate was 91.7% (22/24 cases). mNGS, metagenomic next‐generation sequencing; MRSE, methicillin‐resistant staphylococcus aureus.

The combined bacteriology results are described in Fig. 1, with an overall pathogen identification rate of 91.7% (22/24 cases). The success rates for *Staphylococcus*, *Streptococcus*, and the other pathogens were 100% (9/9 cases), 71.4% (5/7 cases), and 100% (6/6 cases), respectively. Both of the failed cases were infected with *Streptococcus agalactiae*.

## Discussion

The management of acute PJI presents a great challenge to orthopaedic surgeons. The extraction of the implant and the performance of one‐stage or two‐stage revisions may represent a difficult choice for both the patients and surgeons. Thus, DAIR treatment, which retains the protheses, remains the mainstay treatment for acute PJI. However, its efficacy has been debated during the past few decades. Several studies have reported an overall success rate of less than 50%^10^, ^11^, ^12^, ^13^, ^14^, ^15^, ^16^, ^17^, ^18^ (Table 3). A systematic review by Romano *et al*., including 14 articles and 710 acute PJI cases, observed that the success rate of DAIR was as low as 46%^19^. At the recent 2018 International Consensus Meeting (ICM), 96% of the delegates agreed that the effectiveness of DAIR is still unclear, and the treatment decision must be made on a case‐by‐case basis and must account for underlying medical conditions, infection history, organism characteristics, and surgical history^20^.

**Table 3:** Recent studies reporting the overall success rate of DAIR for the treatment of acute PJI

References	Cases	Primary surgery	Mean follow‐up period	Success rate
Kim *et al*. (2011)^30^	116	TKA	67.2 months	92% (36/39)
Achermann *et al*. (2014)^31^	69	THA/TKA	24.0 months	91.60%
Ottesen *et al*. (2019)^26^	67	TKA	24.0 months	90% (43/48)
Klouche, *et al*. (2011)^32^	12	THA	40 months	75% (9/12)
Westberg *et al*. (2011)^33^	38	THA	48 months	71% (27/38)
Tsukayama *et al*. (1996)^3^	41	THA	45.6 months	68% (28/41)
Cobo *et al*. (2011)^34^	117	THA/TKA	25.0 months	57% (67/117)
Marculescu *et al*. (2006)^18^	99	THA/TKA	23.3 months	47% (46/99)
Zmistowski *et al*. (2011)^10^	103	THA/TKA	36 months	46% (47/103)
Odum *et al*. (2011)^14^	150	THA/TKA	NA	31% (46/150)
Koyonos *et al*. (2011)^13^	138	THA/TKA	54 months	31% (16/52)
Crockarell *et al*. (1998)^16^	42	THA	75.6 months	26% (6/23)

DAIR, debridement, antibiotics, and implant retention; PJI, periprosthetic joint infection; NA, not available; THA, total hip replacement; TKA, total knee replacement.

This study represents a single‐surgeon series reporting the outcomes of standardized DAIR procedures for the treatment of acute PJI. The main finding is that DAIR has an overall high success rate for the treatment of acute PJI, despite the universal low success rate that has been reported in the published literature. However, after an in‐depth review, we observed great heterogeneity in the included population. In some reports, the polyethylene inserts/femoral heads were not exchanged during DAIR. This may lead to incomplete debridement, as the surgeon would have no access to the posterior joint capsule and the bilateral gutters. Along with the biofilm adhered to the insert/femoral head, the kept insert/femoral head would definitely increase the bioburden of infection. In addition, surgeons would have the opportunity to clean the tibial/femoral component once the insert or head is removed. Second, in some other reports, surgeries were performed by different surgeons with various experience levels and surgical skills. DAIR is an urgent surgery that requires radical debridement of all the infected tissues, and a standardized operating procedure should be determined. Finally, for most of the reported studies, the positive rates of the cultures were not high. This may increase the likelihood of failure because no pathogen‐specific antibiotics could then be used. In our case series, all the DAIR procedures were performed by a single experienced senior surgeon using a standardized surgical procedure. The culture positive rate was as high as 90% (following a strictly standardized culture protocol); with the aid of the mNGS technique, we were able to identify pathogens in over 90% of the patients and chose pathogen‐specific antibiotics according to the drug‐resistant results.

Next‐generation sequencing, especially mNGS, is an evolving technology that is widely applied in clinical diagnoses^21^. mNGS can be used to sequence all nucleic acid fragments in a clinical sample, thus enabling the use of the bioinformatics method to obtain microbial sequences and species information, which then allows for the identification of the pathogen. Recently, and owing to the substantial cost reduction, mNGS has also been increasingly applied for the diagnoses of bone and joint infections^22^. Our previous reports have demonstrated that mNGS has the potential to identify pathogens in cases with negative culture and even in cases with antibiotic treatment or in cases with rarely seen pathogens^23^, ^24^, ^25^. In this limited cohort of patients, mNGS was also used to identify additional microorganisms in 2 cases, which added further evidence that mNGS may serve as a potential tool for quickly diagnosing PJI. However, more studies are warranted to prove its true efficacy. In our current practice, mNGS has been used as a routine diagnostic tool for suspected bone and joint infections.

Our second finding was that the use of DAIR could be considered even in patients whose symptoms were sustained for over 1 month. In our case series, 5 patients presented with the onset of symptoms between 4 and 8 weeks. These patients were all managed with the use of DAIR, and we observed no infection relapse during a minimum 1‐year follow‐up. A similar study was reported by Ottesen *et al*., which demonstrated a high success rate of 88% in patients within 42 days 26. Nonetheless, we have had no experience in managing patients whose symptoms have been evident for over 3 months. Ottesen reported 10 such patients with DAIR, with a success rate of 60%, which was encouraging^26^. Further evaluations are necessary to determine whether DAIR can be performed to treat delayed or chronic PJI cases. In addition, it must be remembered that the definition of “acute” itself is not clear, partly because it is difficult to precisely determine the onset of symptoms, and partly because, according to some authors, the onset of symptoms can occur in less than 4 weeks, whereas other authors have suggested that the onset can occur in less than 6 weeks or even in 3 months. It appears that a more valid classification system is warranted to guide treatment. In a recent study, Pellegrini *et al*. (2019) suggested that a novel topographic staging algorithm can be used for PJI 5.

The third finding of our study was that the use of DAIR had a high success rate for staphylococcal infections. All 3 cases with *Staphylococcus aureus* infections were successfully treated, and even for methicillin‐resistant staphylococcus, as was observed in our 9 MRSE infections, the use of DAIR completely eradicated the infections. These data were similar to those in a report by Leijtens *et al*., which demonstrated a 78% success rate (14/18 cases) for acute staphylococcal PJI 27. Staphylococcal infections have been viewed as being a risk factor for failed DAIR, as the literature has reported a high failure rate, ranging from 45% to 76% (Table 4). One possible explanation is that staphylococcus more easily forms a biofilm on the surface of the implants, as well as its drug‐resistance to antibiotics 3. Novel materials, such as α‐tocopheryl acetate and α‐tocopheryl phosphate, have been observed to be able to prevent biofilm formation by *Staphylococcus aureus*, which is encouraging, as these materials may act as promising tools to be incorporated into the DAIR procedure^27^. For streptococcal infections, our limited data demonstrated a generally satisfactory success rate of 71.4% (5/7 cases), which was still higher than the data from a multicenter study (35%, 11/31 cases)^14^.

**Table 4:** High failure rate of acute staphylococcal infection treated with DAIR

References	Cases	Microorganism	Follow‐up	Failure rate
Koyonos *et al*. (2011)^13^	84	*Staphylococcus*	54 months	69% (58/84)
Odum *et al*. (2011)^14^	150	*Staphylococcus*	7.8 months	methicillin‐sensitive 72% (48/67); methicillin‐resistant 76% (22/29)
Lora‐Tamayo *et al*. (2013)^35^	345 (81 MRSA)	*Staphylococcus aureus*	NA	45%
Azzam *et al*. (2010)^11^	104	*Staphylococcus*	68 months	64% (40/59)
Gardner *et al*. (2011)^36^	44 TKA	*Staphylococcus*	60 months	*Staphylococcus aureus* (71%); *Staphylococcus epidermidis* (29%)
Konigsberg, *et al*. (2014)^37^	42 hematogenous infections	*Staphylococcus*	56 months	50% (8/16) *vs* non‐*Staphylococcus* 4% (1/26)

DAIR, debridement, antibiotics and implant retention; TKA, total knee arthroplasty; NA, not Available.

Another finding of this study concerned the antibiotic regimen. In our series, IV antibiotics were generally used for 2 weeks, followed by a 4‐week use of oral antibiotics, regardless of cases of hip or knee infections. The 2013 guidelines from the Infectious Diseases Society of America (IDSA) have recommended the use of IV antibiotics for 2–6 weeks, followed by the use of oral antibiotics for 3 months for hip infections and for 6 months for knee infections for treating acute PJI 28. Nonetheless, the optimal duration of antibiotic usage has been controversial. Various studies have observed similar infection eradication rates between short‐term antibiotic regimens and long‐term antibiotic regimens^20^. In addition, a recent multicenter randomized trial demonstrated that oral antibiotic therapy was noninferior to intravenous antibiotic therapy when used during the first 6 weeks for the treatment of complex orthopaedic infections^29^. Long‐term antibiotic regimens can result in several adverse effects to patients, and our study suggests that a short‐term antibiotic regimen (2 weeks of IV antibiotic treatment, plus 4 weeks of oral antibiotic treatment) can be considered for acute PJI. More randomized controlled trials are warranted to address this issue.

There were several limitations of this study that need to be acknowledged. First, similar to other reports, this was a retrospective study that lacked comparative controls and that had a limited sample size. Second, the follow‐up period was relatively short, and it is possible that some slow, low‐virulence infections may have presented themselves at a later time period than our mean 2‐year follow‐up period. The long‐term treatment outcomes remain to be evaluated.

### *Conclusion*

Debridement, antibiotics, and implant retention has a high success rate for the treatment of acute PJI. In selected patients whose symptoms have been sustained for over 4 weeks, DAIR can be considered as a treatment option. DAIR also has a high rate of success for staphylococcal infections.
